# Chitotriosidase as a biomarker of cerebral adrenoleukodystrophy

**DOI:** 10.1186/1742-2094-8-144

**Published:** 2011-10-20

**Authors:** Paul J Orchard, Troy Lund, Wes Miller, Steven M Rothman, Gerald Raymond, David Nascene, Lisa Basso, James Cloyd, Jakub Tolar

**Affiliations:** 1 Department of Pediatrics, Program in Blood & Marrow Transplantation, University of Minnesota, Minneapolis, USA; 2 Department of Pediatrics, Program in Neurology, University of Minnesota, Minneapolis, USA; 3 Department of Neurology, Kennedy Krieger Institute, Baltimore MD, USA; 4 Department of Diagnostic Radiology, University of Minnesota, Minneapolis, USA; 5 Department of Experimental and Clinical Pharmacology, Center for Orphan Drug Research, University of Minnesota, Minneapolis, USA

**Keywords:** biomarker, adrenoleukodystrophy, neuroinflammation, chitotriosidase

## Abstract

**Background:**

Adrenoleukodystrophy (ALD) is an X-linked peroxisomal disorder characterized by the abnormal beta-oxidation of very long chain fatty acids (VLCFA). In 35-40% of children with ALD, an acute inflammatory process occurs in the central nervous system (CNS) leading to demyelination that is rapidly progressive, debilitating and ultimately fatal. Allogeneic hematopoietic stem cell transplantation (HSCT) can halt disease progression in cerebral ALD (C-ALD) if performed early. In contrast, for advanced patients the risk of morbidity and mortality is increased with transplantation. To date there is no means of quantitating neuroinflammation in C-ALD, nor is there an accepted measure to determine prognosis for more advanced patients.

**Methods:**

As cellular infiltration has been observed in C-ALD, including activation of monocytes and macrophages, we evaluated the activity of chitotriosidase in the plasma and spinal fluid of boys with active C-ALD. Due to genotypic variations in the chitotriosidase gene, these were also evaluated.

**Results:**

We document elevations in chitotriosidase activity in the plasma of patients with C-ALD (n = 38; median activity 1,576 ng/mL/hr) vs. controls (n = 16, median 765 ng/mL/hr, p = 0.0004), and in the CSF of C-ALD patients (n = 38; median activity 4,330 ng/mL/hr) vs. controls (n = 16, median 0 ng/mL/hr, p < 0.0001). In addition, activity levels of plasma and CSF chitotriosidase prior to transplant correlated with progression as determined by the Moser/Raymond functional score 1 year following transplantation (p = 0.002 and < 0.0001, respectively).

**Conclusions:**

These findings confirm elevation of chitotriosidase activity in patients with active C-ALD, and suggest that these levels predict prognosis of patients with C-ALD undergoing transplantation.

## Introduction

Adrenoleukodystrophy (ALD) is an X-linked, peroxisomal disorder of very long chain fatty acid (VLCFA) metabolism, resulting in the accumulation of VLCFA in the adrenal gland, testes and brain. The disease frequency is approximately 1 in 17,000 males, and has been reported to be similar in distribution across ethnic and racial groups [1,2]. The capacity to metabolize VLCFA, a reaction that normally takes place in the peroxisome, is impaired in patients with X-ALD due to defects in the ABCD1 gene encoding a peroxisomal membrane protein designated ALDp. A large number of genetic mutations have been identified as causing disease, and there is substantial clinical variability within kindreds despite a conserved genotype [2,3].

The most severe phenotype of ALD is the cerebral form (C-ALD), which is observed in approximately 40% of children affected by ALD. The median age of clinical onset is 7 years. A characteristic finding associated with C-ALD is inflammation of the white matter of the brain, with changes suggesting active oxidative damage thought to be due to the inflammatory process [4]. The disease is associated with progressive demyelination, and once initiated, generally leads to a vegetative state or death within several years of onset. The only available therapy shown to provide long-term stabilization of C-ALD is allogeneic hematopoietic stem cell transplantation, although there is an interest in the development of gene therapy [5]. At this time, the mechanism by which transplantation arrests the disease process is incompletely understood. It is thought to be due, at least in part, to modulation of the neuroinflammatory process. Given the risks associated with transplantation, the current standard of care for neurologically asymptomatic patients is to monitor them prospectively for cerebral involvement by scheduled MRI imaging. If white matter changes with gadolinium enhancement are observed, providing evidence of active inflammation and progression, transplantation should be expediently performed.

Currently, there is no clear means of determining which patients with ALD are likely to develop C-ALD. In addition, for patients with symptomatic disease considering transplantation, predicting outcome is very difficult. While these advanced patients may remain relatively neurologically stable undergoing transplantation, in many cases dramatic progression is observed in the peri-transplant period. The Loes MRI severity scoring system was established to quantify the extent of white matter changes [6], but this does not closely correlate with clinical findings. The rate of progression may be determined with serial MRI scans. However, if transplantation is being considered for patients with active, extensive disease it is impractical to wait a period of months to assess this, as any delay could increase the risk of transplantation and worsen outcomes. Clearly, means of assessing the rate of progression of C-ALD, and thereby potentially establishing prognosis, are necessary. It is possible that inflammatory biomarkers correlate with the rate of deterioration, but meaningful means of accomplishing this have not been established.

Chitotriosidase (CHIT), an enzyme produced by activated monocytes and macrophages, appears to correlate with the extent of disease in Gaucher, and in other neurodegenerative diseases [7-9]. As monocytes and macrophages have been shown to be present within cellular infiltrates in C-ALD [4,10], we measured CHIT activity in the plasma and spinal fluid in boys with C-ALD referred to the University of Minnesota for consideration of transplantation. In addition to the analysis of enzyme activity, we performed PCR analysis of the chitotriosidase gene, as approximately 35% of individuals have a 24 base insert in exon 10 that results in decreased enzyme activity [11]. In these studies, we identified highly significant elevations of chitotriosidase activity in both the plasma and spinal fluid of boys with active C-ALD. Enzyme activity in samples obtained prior to transplantation are shown to be correlated to disease severity as assessed by the MRI severity scoring system, as well as to the functional status of the boys prior to and after transplantation.

## Patients and Methods

### Demographics of Patients Studied

Patients in these studies were confirmed to have ALD based on VLCFA profiles, and had MRI scans documenting white matter changes and gadolinium enhancement consistent with active cerebral disease. Consents for blood and spinal fluid research specimens were obtained in association with the consent for transplantation, as lumbar puncture is performed during the pre-transplantation evaluation. However, not all patients were treated by transplantation, as in some cases advanced patients were not thought to be appropriate to offer transplantation. Samples on other affected individuals, including C-ALD patients that did not proceed to transplantation, or from controls undergoing scheduled phlebotomy and/or a lumbar puncture (LP) for other clinical reasons, were obtained under another Institutional Review Board (IRB) protocol. The control population consisted predominately of children with acute leukemia without cerebral involvement, undergoing lumbar puncture as part of their scheduled chemotherapy or surveillance monitoring in accordance with established treatment protocols. To alleviate concerns about this population serving as a control group, none of these patients had active disease at the time samples were collected. Of the 42 C-ALD patients studied, in one case a plasma sample was not obtained, and in another and no spinal fluid was available. The median age of ALD patients entered on this study was 8.6 years old, (range 4 to 14 years of age). The median age of the 17 controls was 5.6 years, with a range of 2-18 years of age.

### Chitotriosidase Enzymatic Assay

Chitotriosidase activity was measured using a modification of the technique described by Sotgui et al, 2006 [12]. Blood or CSF samples were diluted in buffer [10 mM Tris-HCL, 15 mM NaCL, pH 7.5], and 20 μl aliquots of these dilutions were incubated with 20 μl of 22 μM 4-methylumbelliferyl-beta-D-N,N',N'-triacetyl-chitotriose (MUTAC; Sigma, St. Louis, MO; Cat. #M5639) in 0.5 M citrate-phosphate buffer, pH 5.2, in 0.1% Albumin (Sigma, Cat. #A8412) pre-coated 96 well plates (Fisher; Pittsburgh, PA; Cat. #353072) for 1 hour at 37°C. The reaction was stopped after 1 hour with 250 μl 0.5 M Na_2_CO_3_-NaHCO_3 _buffer, pH 10.7. Enzymatic cleavage of MUTAC produces a fluorescent product, 4-methylumbelliferone (4-MU), which was read on a Molecular Devices, SpectraMAX Gemini fluorometer with 365 nm excitation and 450 nm emissions. The comparison of relative fluorescent units (RFU) with CHIT standards (R&D, Minneapolis, MN; Cat. #3559-GH) ranging from 0.4-12.5 ng/well allowed calculation of CHIT activity, which is expressed as nmoles 4-MU generated/mL of sample (plasma, CSF) per hour (hr).

### Chitotriosidase Genotypic Analysis by PCR

The chitotriosidase gene is comprised of 12 exons on chromosome 1q31-q32, spanning 20 kb. In approximately 35% of the population a 24 base duplication is present in exon 10, resulting in the activation of a 3' splice site and a 87 nucleotide deletion, decreasing CHIT activity by 50%. Approximately 5% of individuals are homozygous for this mutation, resulting in the absence of enzyme activity. We developed a PCR assay to document these genotypes due to their importance in assessing CHIT activity. Genomic DNA was isolated from leukocytes (Gentra Puregene Blood Kit, Qiagen, Valencia, CA; Cat. #158467). The sense oligonucleotide was designed to anneal within the intron (5'-CTGTCCAGAAGAGGTAGCCA-3') and the antisense primer within exon 10 (5'- GGAGAAGCCGGCAAAGTC-3') to amplify band sizes of 160 bp (wild-type gene) and/or 184 bp (insertion). This allows differentiation of subjects homozygous for the wild-type genotype from heterozygotes, and from those homozygous for the 24 base deletion. The PCR reaction was performed with 125 ng of genomic DNA, 200 μM dNTP's, 3 mM MgCl_2_, 500 nM oligonucleotides, and 1 unit of Taq at 94°C (1 min), 56.2°C (30 sec) and 72°C (30 sec) for 30 cycles. Using this information, we excluded 3 ALD patients shown to be homozygous for this insertion; the lack of chitotriosidase activity was documented in all 3 cases. For those patients heterozygous for this duplication, chitotriosidase activity is reported as twice the value determined by the assay to compensate for the anticipated loss in activity, as has been done in other investigations [13-15].

### Patient Assessments

The MRI scans were evaluated by a single neuroradiologist (DN) and scored according to the Loes scoring system, as previously described [16]. To define clinical severity, we used a scoring system previously describe by the Moser and Raymond (Table 1) [17]. In patients assessed more recently this was done prospectively. Alternatively, the scoring was performed retrospectively from neurologic evaluations provided in patient records. As many patients came from a distance and could not return for routine one-year evaluations at a designated time, data considered as the 1-year evaluation for both Loes and Moser-Raymond scoring was that captured closest to 1 year post transplant, considering data obtained at least 100 days after transplant and not greater than 18 months after transplant. The change in Loes and functional scores were assessed by subtracting the baseline scores prior to transplantation from the 1-year time point, and are listed as the "Delta" for both the Loes and functional scoring systems.

**Table 1:** Moser-Raymond Severity Scoring System: The scoring system used in this analysis to determine the clinical status of patients with ALD was previously developed by Moser and Raymond [17]

Hearing/auditory processing problems 1	1
Aphasia/apraxia	1

Loss of communication	3

Vision impairment/fields cut	1

Cortical blindness	2

Swallowing difficulty or other central nervous system dysfunction	2

Tube feeding	2

Running difficulties/hyper-reflexia	1

Walking difficulties/spasticity/spastic gait (no assistance)	1

Spastic gait (needs assistance)	2

Wheelchair required	2

No voluntary movement	3

Episodes of incontinency	1

Total incontinency	2

Nonfebrile seizures	1

**Possible Total**	**25**

A score for each patient was established at baseline (prior to transplantation) and at 1 year following transplantation. The difference (delta) is presented as the clinical neurologic progression to one year after transplant in Figures 3 and 4.

### Statistical methods

Differences in chitotriosidase activity in plasma and spinal fluid between patients and controls were determined using the unpaired t test with Welch's correction. Linear regression analysis was performed to determine correlations between chitotriosidase activity and outcomes, including Loes and functional scores. The 2-tailed Pearson's correlation was used in determining correlations of chitotriosidase activity in CSF and plasma.

## Results

### Determinations of Chitotriosidase Genotype

We determined the chitotriosidase genotype of individuals in addition to the activity of chitotriosidase. DNA was available for 41 of the 42 ALD patients, of whom 22 (53.7%) were homozygous for the wild-type genotype, 16 (39%) were heterozygous for the 24 bp duplication, and 3 (7.3%) were homozygous for the duplication. This distribution is similar to prior observations [11,13,18,19]. In the control population, 17 plasma and spinal fluid samples were available, with DNA samples on 16 of these controls. One control subject (6.3%) was shown to be homozygous for the duplication, four (25%) were heterozygous for the duplication, and 11 (68.7%) were homozygous for the wild-type genotype. In the two cases where DNA was not available (one ALD patient and the one control), chitotriosidase activity was confirmed; these samples were assumed to be associated with a wild-type genotype. In all cases, (three ALD patients and one control) shown to be homozygous for the duplication, chitotriosidase testing was performed, and in all cases no activity was measurable. Each of these cases was excluded from further analysis. Therefore, chitotriosidase activity could be assayed on the plasma and spinal fluid of 38 patients with ALD and 16 controls.

### Determinations of Plasma and CSF Chitotriosidase Activity

**Figure 1:**
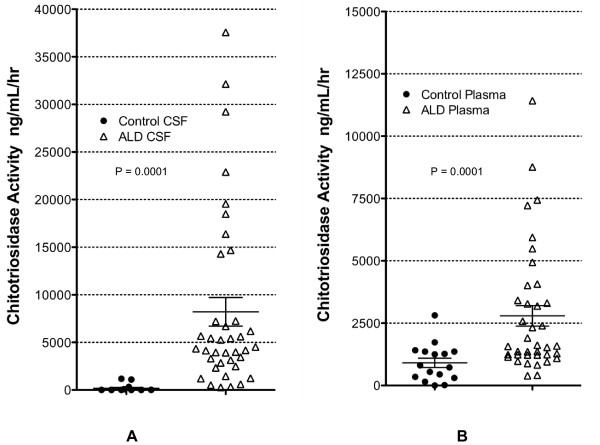
**Chitotriosidase Activity is Elevated in Patients with ALD: **Chitotriosidase activity was evaluated in the spinal fluid (Figure 1A) and plasma (Figure 2B) of patients with cerebral ALD or controls.  There were 38 ALD patient samples and 16 controls represented in each group

Cerebral spinal fluid samples were available for 16 control subjects and 38 patients with C-ALD shown not to be homozygous for the 24 base duplication resulting in a lack of activity. In the control population, the median CHIT activity in the spinal fluid was 0 ng/mL/hr (mean 168, range 0 to 1,180 ng/mL/hr). In the C-ALD patients, median activity in the spinal fluid was 4,424 ng/mL/hr (mean 8,212, range 276 to 37,564 ng/mL/hr; Figure 1A; p < 0.0001).

**Figure 2:**
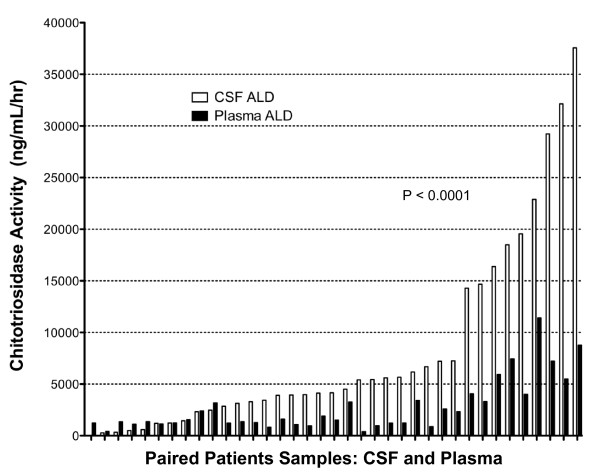
**Chitotriosidase Activity Correlates in C-ALD Plasma and Spinal Fluid: **. For the 37 patients with cerebral ALD for which both plasma and spinal fluid were available, the relative activity for both are depicted.  For each patient, Statistical significance related to correlations of the 2 groups is shown (Pearson two-tailed analysis).

Plasma samples were available for 16 control subjects and 38 patients with C-ALD. The median activity in the control plasma samples was 765 ng/mL/hr, with a mean of 908 and a range of 0 to 2,812 ng/mL/hr. By comparison, median plasma C-ALD activity was 1,576 ng/mL/hr (mean 2,793, range 390 to 11,420 ng/mL/hr; Figure 1B; p = 0.0001). For those patients with both plasma and CSF samples, the relative plasma and CSF chitotriosidase activity for each individual patient is shown (Figure 2). The correlation of the CSF and plasma activity levels is < 0.0001.

### Correlations of Chitotriosidase Activity with Loes Score

**Figure 3:**
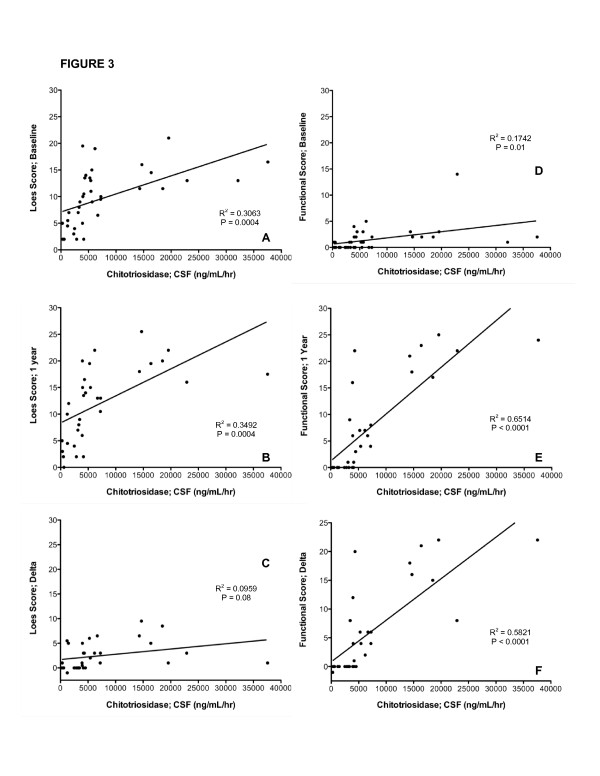
Spinal Fluid Chitotriosidase Determinations Are Associated with MRI and Functional Scores For ALD patients with cerebral disease, the correlation of CSF chitotriosidase activity prior to transplantation and the baseline Loes MRI severity score (Fig 3A), the Loes score 1 year post transplantation (3B) and the relative increases in the Loes score from baseline to 1 year after transplantation (Loes Score; Delta; Fig 3C) are presented.  The correlation of CSF chitotriosidase activity to the Moser/Raymond functional score (Table 1) prior to transplantation (Fig 3D), at 1 year after transplantation (Fig 3E) and in regards to the change in the functional score from baseline to 1 year after transplant (Functional Score; Delta; Fig 3F) are shown.

**Figure 4:**
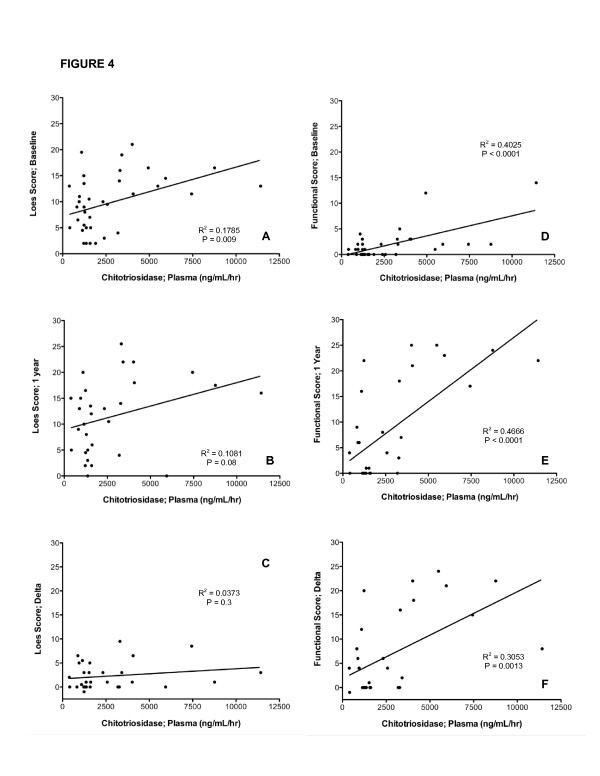
**Plasma Chitotriosidase Determinations Are Associated with MRI and Functional Scores:** For ALD patients with cerebral disease, the correlation of plasma chitotriosidase activity prior to transplantation and the baseline Loes MRI severity score (Fig 4A), the Loes score 1 year post transplantation (4B) and the relative increases in the Loes score from baseline to 1 year after transplantation (Loes Score; Delta; Fig 4C) are presented.  The correlation of plasma chitotriosidase activity to the Moser/Raymond functional score prior to transplantation (Fig 4D), at 1 year after transplantation (Fig 4E) and in regards to the change in the functional score from baseline to 1 year after transplant (Functional Score; Delta; Fig 4F) are shown.

We investigated whether plasma and spinal fluid chitotriosidase activity correlated with extent of disease based on Loes MRI scores. When CSF (Figure 3A) and plasma (Figure 4A) chitotriosidase activity is analyzed in relationship to the pre-transplant (baseline) Loes score, there was a statistically significant correlation (p = 0.004 and 0.009, respectively). We also evaluated the correlation between the pre-transplant chitotriosidase activity and Loes score at one year, and also in the change in Loes score (Delta score) before and one year after transplant to determine whether chitotriosidase activity pre- transplant is predictive of a change in Loes score. We found that the spinal fluid chitotriosidase significantly correlated with one-year post transplant Loes score (Figure 3B; p = 0.0004), but not with change in Loes score (Figure 3C). The plasma chitotriosidase activity failed to correlate with either the Loes score one year post transplant or the change in Loes score (Figure 4B and 4C).

### Correlations of Chitotriosidase Activity with Functional Score

The functional scores of the patients prior to and one year post-transplant were subsequently analyzed. The change in functional score was determined by subtracting the score at 1 year from the baseline score as a measure of clinical disease progression. The correlation of CSF chitotriosidase activity to the baseline functional score is provided in Figure 3D; this correlation is significant (p = 0.01). Importantly, the correlation between chitotriosidase activity in the spinal fluid prior to transplantation proved even more significant in the linear regression analysis of the neurologic functional score 1 year following transplantation (p < 0.0001; Figure 3E) and the change in the neurologic functional score from baseline to 1 year post transplantation (p < 0.0001; Figure 3F). When this same analysis is performed investigating the plasma chitotriosidase activity, the correlation was high in regard to the baseline functional score (p < 0.0001; Figure 4D) and the one-year post transplantation functional score (p < 0.0001; Figure 4E) but less highly correlated with the change in functional score (p = 0.0013; Figure 4F).

## Discussion

We report for the first time highly significant elevations of chitotriosidase activity in patients with cerebral ALD. We reasoned that the chitotriosidase activity would be elevated because of the previously documented presence of monocytes and macrophages in the central nervous system of individuals with cerebral ALD [10,20]. We demonstrate that CHIT activity is elevated in both plasma and spinal fluid, although levels are in general much higher in CSF. Patients with higher CSF activity also tend to have higher activity in the plasma (Figure 2). We next asked whether CHIT activity in the CSF and plasma correlated to the extent of disease as defined by the MRI severity score described by Loes [16,21]. In these analyses, both the CSF (Figure 3A) and plasma (Figure 4A) activity were significantly correlated to the "baseline" MRI scores, which would be closest in time to when the samples were obtained (p = 0.0004 and 0.012, respectively). The correlation of chitotriosidase activity was also analyzed in relationship to the MRI severity scores at 1 year following transplant. In the case of plasma activity (Figure 4B), this correlation was not significant (p = 0.08), while the CSF activity was highly correlated to the Loes score at one year post transplant (Figure 3B, p = 0.0004). When the correlation of CHIT activity to disease progression by MRI (Loes score; Delta) is analyzed, neither plasma nor CSF activity values were significantly correlated to the change in Loes score (Figures 3C and 4C).

The majority of C-ALD patients transplanted early in the course of their disease have minimal or no subsequent clinical manifestations. In contrast, patients with more advanced disease often exhibit substantial disease progression post transplant [22]. To better assess these functional parameters, we used the Moser-Raymond scale (Table 1). The functional status of the patients was determined prior to transplantation and at 1 year after the transplant. Evidence of clinical disease progression may be defined as the difference in these scores. Chitotriosidase activity was shown to be highly correlated with the pre-transplant functional score, but more importantly, also to the clinical status of the patients post transplantation. This is apparent when chitotriosidase activity is assessed in relation to the 1-year scores (CSF and plasma; p < 0.0001) and in relationship to the change in functional status (p < 0.0001 and < 0.0013 in CSF and plasma, respectively).

The ability to better establish prognosis in patients being considered for allogeneic transplantation is of great importance. Based on our experience and those of others, patients early in the course of cerebral disease are very likely to achieve disease stabilization without significant clinical deterioration. In contrast, for patients with more advanced disease there is great variation in outcomes after transplantation, with relatively mild progression observed in some patients and dramatic deterioration in others. Standard means of assessing these patients include MRI, neurologic examination, neuropsychological testing and potentially functional assessments. The data presented in this study suggests that chitotriosidase determinations can provide important prognostic information, and may allow physicians and families to make a much more informed decision on whether transplantation is the best course of action.

Elevated chitotriosidase activity has been described in other neurologic disorders, including stroke and multiple sclerosis (MS) [12,23-25]. While material that appears similar to chitin was identified in Alzheimer's disease, it was not shown to be present in multiple sclerosis [26]. In the case of ALD the etiology cannot be directly assessed, but it seems likely that the increases in chitotriosidase activity are likely related to inflammation, particularly since the elevations are also apparent in the plasma of patients with ALD. Interestingly, while chitotriosidase is elevated in the CSF in both relapsing-remitting and primary progressive MS, it is not elevated in the plasma [25]. This is in contrast to our findings in ALD. This may suggest that the inflammation in ALD is more systemic in nature than that observed with MS.

These findings suggest other important questions that cannot be addressed in this study. Is chitotriosidase activity related directly to damage within the CNS, or is it merely a biomarker of disease? Is there any difference in the distribution of the chitotriosidase 24 base insert in exon 10 in ALD and the general population? From our studies it would appear not, but this could only be addressed with a larger population of patients. Would determinations of plasma or spinal fluid chitotriosidase activity improve our ability to predict which patients diagnosed with ALD are likely to progress to C-ALD? In addition, is chitotriosidase activity increased in patients with adrenomyeloneuropathy, or in female heterozygote "carriers"? Would it be useful clinically in these conditions? Even more intriguing is the possibility that chitotriosidase could prove to be a biomarker for other neurodegenerative diseases that have an inflammatory component, allowing more rational therapeutic decisions. Additional investigations will prove important in further establishing the role of chitotriosidase in ALD and other similar conditions.

## Lists of abbreviations

ALD: Adrenoleukodystrophy; C-ALD: cerebral ALD; CHIT: chitotriosidase; CNS: central nervous system; HSCT: hematopoietic stem cell transplantation; IRB: institutional review board; LP: lumbar puncture; VLCFA: very long chain fatty acids.

## Competing interests

The authors declare that they have no competing interests.

## Authors' contributions

PJO was the Principal Investigator and primary author of the manuscript, and his laboratory was used to perform the laboratory studies. TL collaborated in the design of the laboratory studies, and discussions as to the role of biomarkers in inherited disease with neuroinflammation. WM reviewed clinical information regarding patient outcomes, including the functional scoring system for the patients on this study. SMR reviewed clinical information regarding patient outcomes, including the functional scoring system for the patients on this study (this task was split between WM and SMR). GR, an internationally established expert in peroxisomal disease, established the scoring system used in these investigations and provided assistance with the design and interpretation of the study. DN is a neuroradiologist who read and scored the MRIs used in this analysis. LB is a technician who performed the majority of the studies in the manuscript and wrote the majority of the methods section. JC is a pharmacologist and collaborator in clinical and laboratory studies on adrenoleukodystrophy, and approaches associated with inflammation. JT is a laboratory collaborator who assisted with PCR and chitotriosidase assay development and interpretation. All authors critically reviewed, read, and approved the final manuscript.
